# Supplementary material for: Bridge Nodes between Personality Traits and Alcohol-Use Disorder Criteria: The Relevance of Externalizing Traits of Risk Taking, Callousness, and Irresponsibility
Source: J Clin Med. 2022 Jun 16;11(12):3468. doi: 10.3390/jcm11123468 (PMC9225009; doi:10.3390/jcm11123468)
Supplement: Supplementary file 1 [file jcm-11-03468-s001.zip › jcm-1716674-supplementary.pdf]

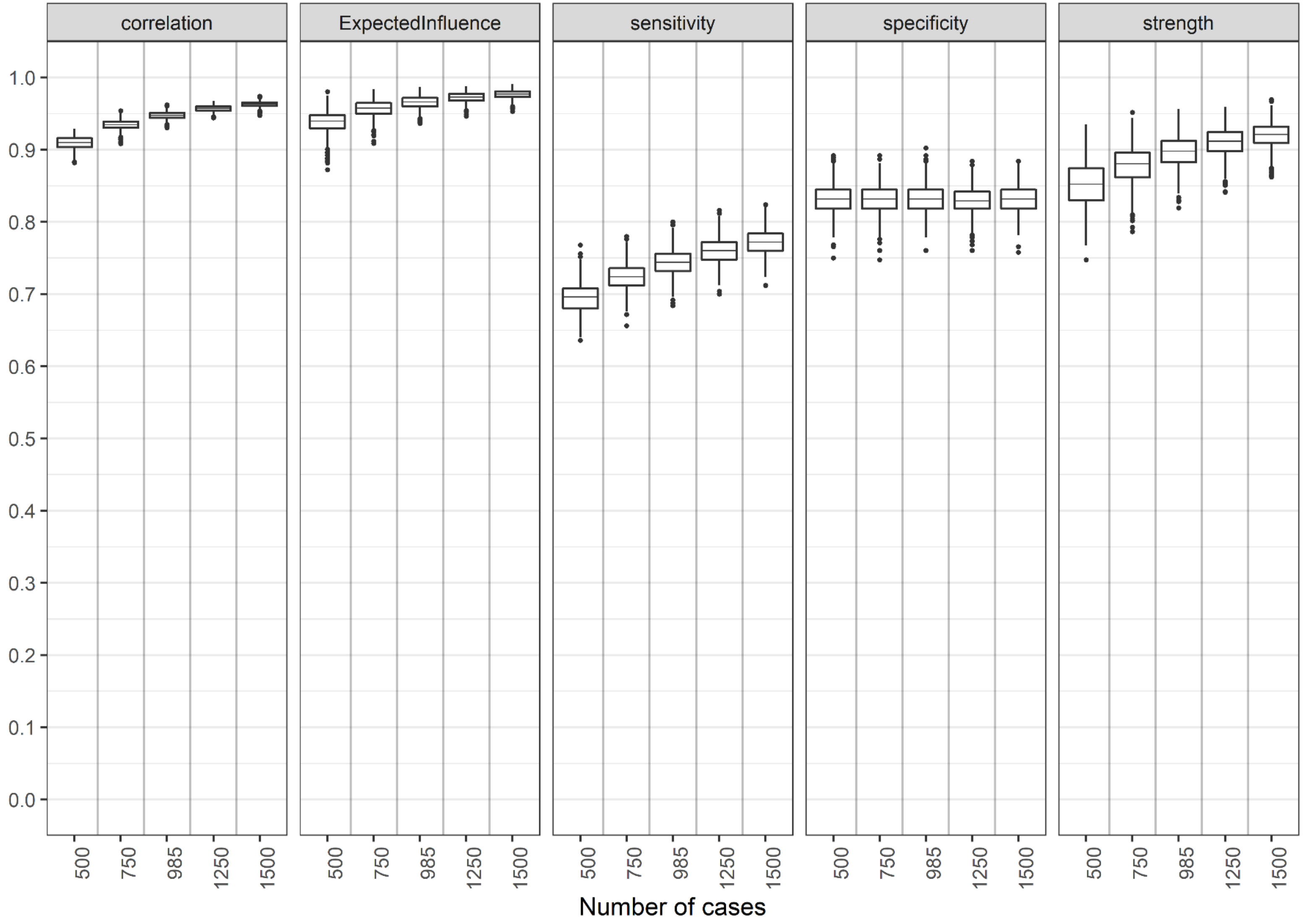

Supplementary Figure S1. Simulation results using the estimated network adjusted to a real network structure for the complete sample (n = 985). From left to right this shows the correlation between the real network and the estimated network. the sensitivity (real positive rate). the specificity (real negative rate). and the correlation of the Strength and Expected Influence measures of centrality between the real network and the estimated network.

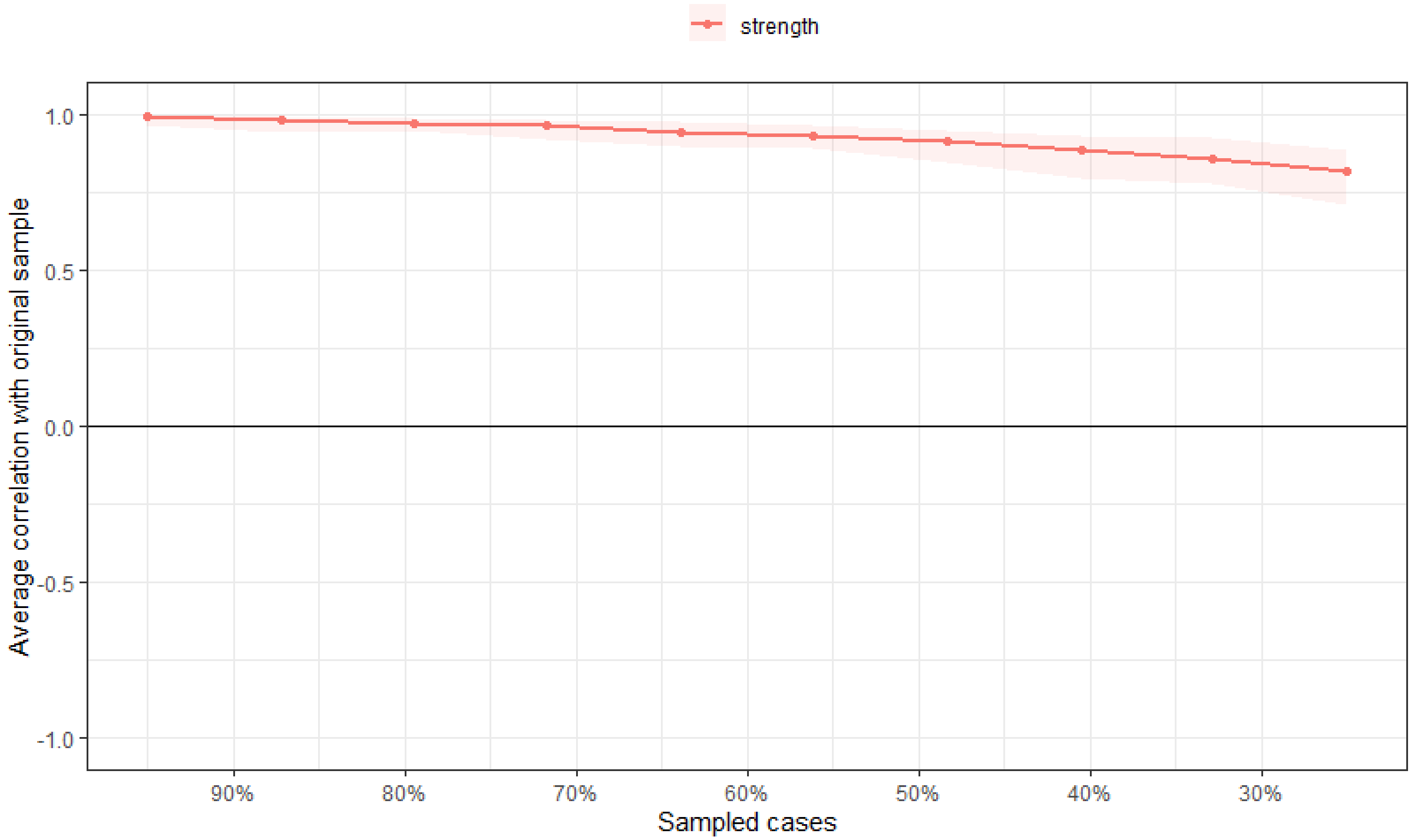

Supplementary Figure S2. Stability plot of the Strength of the network for the complete sample (n =985). La línea representa la media de correlaciones entre un determinado porcentaje de la muestra y el total de la muestra y las sombras representan el área entre el 2.5% y el 97.5% de las correlaciones estimadas.

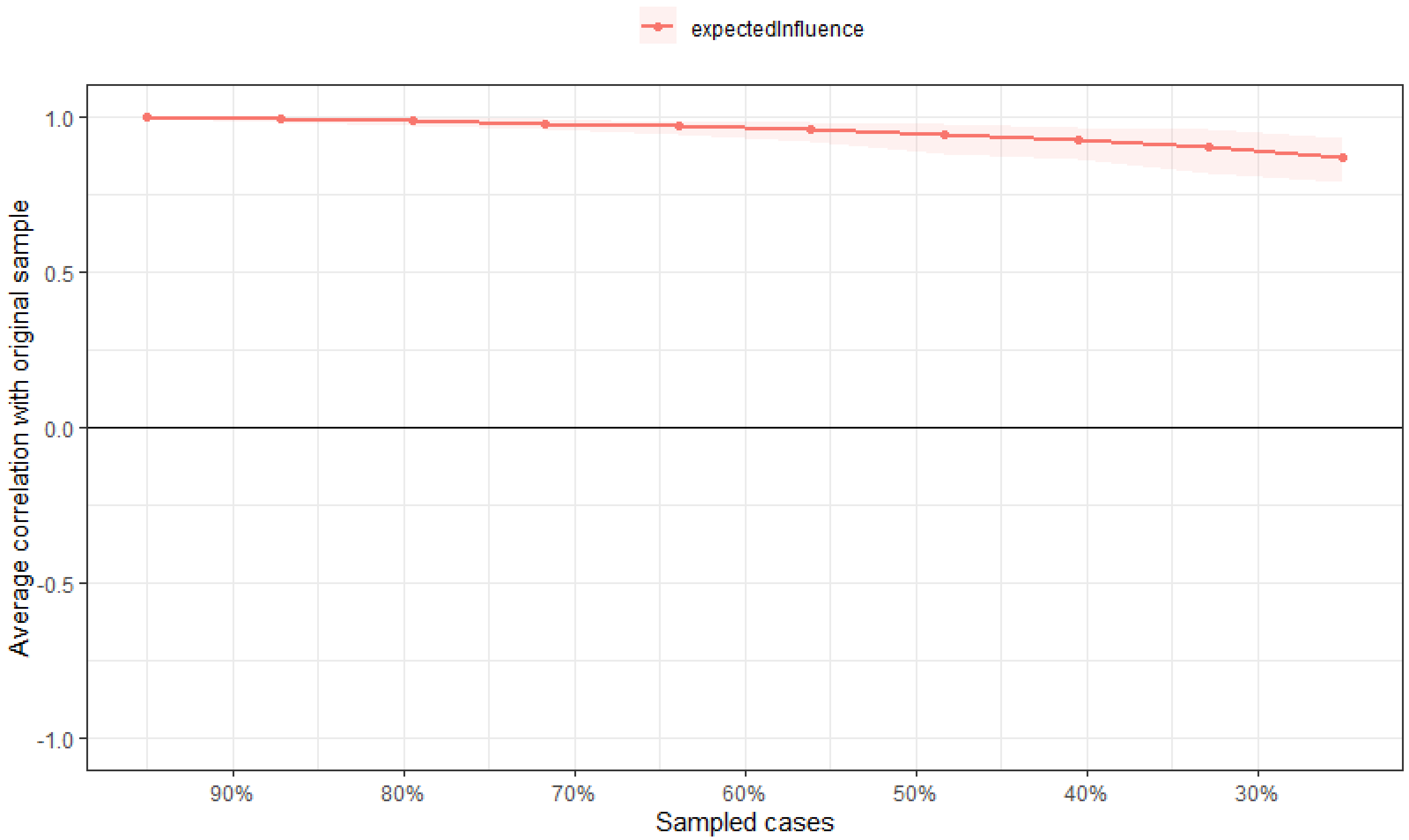

Supplementary Figure S3. Stability plot of the one-step Expected Influence of the network for the complete sample (n = 985). La línea representa la media de correlaciones entre un determinado porcentaje de la muestra y el total de la muestra y las sombras representan el área entre el 2.5% y el 97.5% de las correlaciones estimadas.
